# A morphoscopic exploration of cranial sexual dimorphism among modern South Africans using computed tomography scans

**DOI:** 10.1007/s00414-024-03283-3

**Published:** 2024-07-10

**Authors:** Gabriele Christa Krüger, Richard L. Jantz, Elizabeth van der Walt, Zarina I. Lockhat, Ericka N. L’Abbé

**Affiliations:** 1 Department of Anatomy, Faculty of Health Sciences, University of Pretoria, Private Bag X323, Arcadia, 0007 South Africa; 2 Department of Anthropology, University of Tennessee, Knoxville, TN USA; 3 Department of Radiology, Faculty of Health Sciences, School of Medicine, University of Pretoria, Pretoria, South Africa

**Keywords:** Sex estimation, Cranial variation, Computed tomography, Binary logistic regression, Random forest modelling, Population variation

## Abstract

Continual re-evaluation of standards for forensic anthropological analyses are necessary, particularly as new methods are explored or as populations change. Indian South Africans are not a new addition to the South African population; however, a paucity of skeletal material is available for analysis from medical school collections, which has resulted in a lack of information on the sexual dimorphism in the crania. For comparable data, computed tomography scans of modern Black, Coloured and White South Africans were included in addition to Indian South Africans. Four cranial morphoscopic traits, were assessed on 408 modern South Africans (equal sex and population distribution). Frequencies, Chi-squared tests, binary logistic regression and random forest modelling were used to assess the data. Males were more robust than females for all populations, while White South African males were the most robust, and Black South African females were the most gracile. Population differences were noted among most groups for at least two variables, necessitating the creation of populations-specific binary logistic regression equations. Only White and Coloured South Africans were not significantly different. Indian South Africans obtained the highest correct classifications for binary logistic regression (94.1%) and random forest modelling (95.7%) and Coloured South Africans had the lowest correct classifications (88.8% and 88.0%, respectively). This study provides a description of the patterns of sexual dimorphism in four cranial morphoscopic traits in the current South African population, as well as binary logistic regression functions for sex estimation of Black, Coloured, Indian and White South Africans.

## Introduction

The ability of a forensic anthropologist to produce an accurate biological profile to assist in providing a presumptive identification for an unknown individual relies heavily on the methods employed, the statistical analyses incorporated, and the reference data used. This research made use of socio-culturally defined terminology (i.e. Black South African, Coloured South African, Indian South African, and White South African) to refer to the populations, as these terms have been kept for the purposes of redress and identification.

Creating reference databases is influenced by the available reference samples, and in the case of Indian South Africans, skeletal material is severely limited. Until now, no biological anthropological research existed on the cranial variation in the modern Indian South African population and, as such, no reference data was available for estimating the parameters of the biological profile. Furthermore, even though multiple studies have previously assessed the skeletal variation present in Black, Coloured and White South Africans, the reference data was based on sample populations from the previous century that may not be completely representative of the current population variation [1–3].

Cranial sexual dimorphism is population-specific due to the differences in geographical location as well as in the evolutionary influences and experiences of these groups [3, 4]; therefore, the degree of cranial sexual dimorphism and the features that are more or less dimorphic vary between and among populations. Considerable differences in cranial sexual dimorphism between populations invalidate sex estimation techniques when data sources are based on reference populations with different geographical and ancestral origins [3]. Therefore, methods based on one population need to be validated and possibly re-calibrated to improve standards in all populations.

Accurate sex estimation is based on the quantification of the expression of sexual dimorphism and on the degree of sexual dimorphism present in a population [5–7]. In 2015, Krüger and colleagues tested the percent correct obtained when White and Black South Africans were classified using the original Walker [8] equations derived from North American and British samples. The British sample from the St Bride’s collection in London, England, were not only temporally older, but also more gracile than the remaining populations in the Walker [8] study, which has resulted in lower correct classifications for more robust populations, such as modern South Africans [3]. Therefore, the resulting low correct classifications and significant population differences warranted re-calibrating the equations to accommodate the variations in sexual dimorphism noted between Black and White South Africans. However, while the previous research highlighted the importance of populations-specificity, only two of the four larger South African populations were assessed. Skeletal samples for certain populations are either limited to certain skeletal collections (Coloured South Africans mainly found in the Kirsten Collection, Stellenbosch) or are typically not available in any skeletal collection (Indian South Africans). Furthermore, even though Black and White South Africans have been previously studied, the skeletal material housed in South African collections is temporally older and may not be representative of the current population. As secular trends have been shown to affect sexual dimorphism in a population [9, 10], constant re-evaluation of standards used in forensic anthropological analyses are necessary to account for any temporal changes.

To overcome the limitations of finding appropriate, contemporary skeletal material, three-dimensional (3D) virtual cranial models were extracted from head CT scans. Using CT scans as proxies for human bones have shown excellent results. Interlandmark distances from a virtual 3D model are able to provide similar results to linear measurements and digitisation of bones [11, 12]. Therefore, information obtained from the 3D models can be used to explore cranial variation in not only previously unstudied Indian South Africans, but also in contemporary Black, Coloured and White South Africans. This study aimed to explore the cranial sexual dimorphism in morphoscopic traits of modern Black, Coloured, Indian and White South Africans.

## Materials and methods

A total of 408 head CT scans was assessed for the current study with equal sample sizes for males and females of Black, Coloured, White and Indian South Africans (51 per group; Table 1). All scans were obtained from various hospitals in South Africa. The Steve Biko Academic Hospital in Pretoria, Gauteng, the Groote Schuur Hospital in Cape Town, Western Cape, and the Inkosi Albert Luthuli Central Hospital in Durban, KwaZulu-Natal. As certain populations are more populous in certain areas, the scans were obtained from hospitals in areas where the groups are most prevalent. For example, as Coloured South Africans reside mainly in the Western Cape, the scans for the population were obtained from the Groote Schuur Hospital. Furthermore, as Indian South Africans have a larger presence in KwaZulu-Natal, the scans for Indian South Africans were collected from the Inkosi Albert Luthuli Central Hospital. CT scans for Black and White South Africans were widely available throughout the country and thus were collected from all three hospitals. Self-reported or peer-reported population affinity was mostly available with the CT scans, and was recorded in line with the South African Census forms and thus is limited to Black African, Coloured, Indian/Asian, White or Other. As a result of extensive endogamy within most South African population groups, particularly in the Indian South African population, surnames were considered as confirmation of population affinity or for the exclusion of individuals from the sample. While Indian South Africans only make up 2.5% of the South African population, the group is more prevalent in certain suburbs, where they can account for between 55 and 77% of the population [13].

**Table 1:** Distribution and sources of the sample

Population-sex group			Abbreviation	Mean Age	*n*	Source
Black South African males			BM	38.7	34	Groote Schuur Hospital
					17	Inkosi Albert Luthuli Central Hospital
Black South African females			BF	38.7	22	Groote Schuur Hospital
					29	Inkosi Albert Luthuli Central Hospital
**Population group total**					**102**	
White South African males			WM	51.9	16	Steve Biko Academic Hospital
					18	Groote Schuur Hospital
					17	Inkosi Albert Luthuli Central Hospital
White South African females			WF	52.8	28	Steve Biko Academic Hospital
					4	Groote Schuur Hospital
					19	Inkosi Albert Luthuli Central Hospital
**Population group total**					**102**	
Coloured South African males			CM	41.5	51	Groote Schuur Hospital
Coloured South African females			CF	45.5	51	Groote Schuur Hospital
**Population group total**					**102**	
Indian South African males			IM	49.8	51	Inkosi Albert Luthuli Central Hospital
Indian South African females			IF	52.0	51	Inkosi Albert Luthuli Central Hospital
**Population group total**					**102**	
	***Total sample***				***408***	

In order for the crania to be comparable across the various scans, only those with slice thicknesses less than 0.625 mm were used in the current study. Furthermore, isotropy was necessary for the reconstructed 3D model to retain resolution quality in visualisations and to be the most accurate representation of the actual object (cranium). Therefore, in order for all scans to be comparable and isotropic, the scans were re-sliced and saved as isotropic versions.

As this was a retrospective study, the scans for this research were only collected once patients had been scanned for reasons not related to the current project. Only scans where the necessary structures were clearly identifiable and with no injuries disrupting any of the landmarks were included. Furthermore, no scans with any metal objects causing artefacts in imaging acquisitions were retained for further study. All scans were anonymised and no information that could be used to identify the individuals was retained. Only general demographic information, such as population affinity (self- or peer-assigned), sex and age were stored for the individuals.

To prepare the scans for creating the 3D models, the slices were oriented to the Frankfort horizontal plane. Thermo Scientific™ Avizo™ software 9 (license: AVIZO.59,228) was used for alignment and later for the scoring process. The Treatment and Increased Vision for Medical Imaging (TIVMI) program, a software designed to assist anthropology researchers in obtaining precise and reproducible measurements in 3D and medical imaging, was used to segment and volume-render the CT scans to create 3D models of the crania (bone only) [14, 15].

The segmentation involved finding a particular grey value threshold that was able to distinguish the bone from all surrounding structures, such as the air, soft tissues, blood vessels, etc. A modified version of the half-maximum height (HMH) thresholding was used and involved calculating the average of the greyscale values along the boundary transition. The boundary is a row of voxels that illustrate the transition between different tissue types or between the tissue and the air surrounding it [14, 16]. The process was then repeated on 10 randomly selected slices and the mean value of the slices was taken as the threshold for the entire stack [16, 17]. Once segmentation was completed, the segmented materials (i.e., bone) was transformed into the 3D model of a cranium, which was then uploaded into Avizo™ for scoring of the morphological features.

### Morphoscopic traits for sex estimation

Four morphoscopic traits, namely the glabella, mastoid process, nuchal crest and supra-orbital margin were scored on each of the 3D cranial models. Each trait was scored according to a scale of 1–5 using score descriptions that depended on line diagrams published by Walker [8] regarding expression of the trait. While the original Walker [8] method made use of an additional mental eminence trait, many of the head CT scans obtained for this research did not include a mandible, which warranted excluding the trait. The bilateral features were only scored on the left, as testing asymmetry was not within the scope of this study.

### Statistical analyses

The intra- and inter-observer agreement for the scores of the four Walker [8] traits were assessed on 15 crania using a weighted Cohen’s kappa (κ), in order to evaluate consensus within (intra-observer) and between raters (inter-observer) [18]. If both raters were in complete agreement, then κ = 1. If no consistency was found between raters, then κ = 0. Values less than 0.40 represented poor to fair agreement, values between 0.40 and 0.75 indicated medium or good agreement, and values greater than 0.75 indicated excellent agreement between observers [19]. Both the principal investigator and the second observer had more than 10 years’ experience scoring the Walker [8] traits.

In order to evaluate whether a relationship between age and the scores was present, polyserial correlations were conducted for each trait and each population-sex group. Polyserial correlations can be used to assess relationships between ordinal (trait scores) and continuous (age) variables [20].

Frequency distributions of the scores were used to identify general distributions of the scores across the different sexes and populations. Furthermore, the frequencies were used to observe the pattern of sexual dimorphism within Black, Coloured, Indian, and White South Africans. Large differences in the prevalent scores between males and females within a population indicated a greater degree of sexual dimorphism, whereas overlap in the scores indicated limited sexual dimorphism in the group. More scores of 5 also indicated increased robustness, whereas more scores of 1 indicated extreme gracility [3].

Chi-squared tests of independence were used to test for significant differences in the nominal frequencies of the scores among the populations and between the sexes in each population. The test is non-parametric and is thus robust and does not require normality of the data [20–22].

Binary logistic regression was used to predict the probability of the occurrence of an event fitting the data and was used to identify relationships between the binary dependent variable (sex) and the ordinal independent variables (scores of the four morphoscopic traits) [23].

Recent research has also shown the potential of using random forest modelling for sex estimation using morphoscopic traits [24, 25]. Random forest modelling is a flexible machine learning algorithm that operates by constructing a multitude of decision trees through bootstrap aggregating of random training subsets. Overall, model predictions are made by calculating the prediction for each decision tree, then taking the most popular result from all the trees and combining their predictions to determine the best classification rules [25]. A 70% proportion of the sample was used as the training data, while the remaining 30% was used as a test sample. Only the test sample results were presented.

## Results

The results showed excellent intra-observer agreement, with the lowest agreement obtained for the supra-orbital margin (κ = 0.819) and the highest agreement obtained for the mastoid process (κ = 0.909; Table 2). A similar level of agreement was obtained between the principal investigator and the second observer, where the agreement was excellent. However, for the inter-observer agreement, repeatability was lowest for the nuchal crest (κ = 0.769) while also highest for the mastoid process (κ = 0.898; Table 2).

**Table 2:** Intra- and inter-observer agreement for the four morphoscopic traits

	Intra-observer agreement				Inter-observer agreement		
	Cohen’s κ	*p*-value	Landis & Koch (1977) scale		Cohen’s κ	*p*-value	Landis & Koch (1977) scale
glabella	0.871	< 0.001	Excellent agreement		0.811	< 0.01	Excellent agreement
mastoid	0.909	< 0.001	Excellent agreement		0.898	< 0.01	Excellent agreement
nuchal	0.872	< 0.001	Excellent agreement		0.769	< 0.01	Excellent agreement
orbit	0.819	< 0.01	Excellent agreement		0.809	< 0.01	Excellent agreement

To evaluate the relationship between age and the scores of the various traits, polyserial correlations for each trait and each population-sex group were run and ranged from − 0.2913 to 0.2389, indicating very weak negative and positive correlations (Table 3).

**Table 3:** Polyserial correlations to explore the relationship between the various ordinal scores and age for each population-sex group. Bold indicates strongest positive and negative correlations

	Polyserial correlation coefficient			
	glabella-age	mastoid-age	nuchal-age	orbit-age
BM	-0.1419	0.0573	0.0310	-0.1240
BF	0.0527	-0.1626	0.0285	0.0418
CM	0.0662	0.1176	0.1847	-0.0289
CF	**0.2389**	**-0.2913**	-0.0878	-0.2770
IM	-0.0342	0.0845	0.1936	-0.1330
IF	0.1837	-0.1309	0.0041	-0.0206
WM	0.1357	0.1250	-0.0665	-0.0961
WF	0.0250	-0.0328	-0.1187	-0.2317

When the means and standard deviations were explored with only the sexes separated but all the ancestry groups pooled, males presented with overall higher scores (3.44 to 3.66) than females (1.39 to 2.81; Table 4). The standard deviations also indicated higher levels of variation in the males for all four traits when compared to the females (Table 4).

**Table 4:** Frequencies, means, and standard deviations (SD) of the scores for males and females for each of the four morphoscopic traits

Glabella								
	1	2	3	4	5		mean	SD
M	18	56	77	81	72		3.44	1.20
F	206	82	12	3	1		1.39	0.65

Mastoid								
	1	2	3	4	5		mean	SD
M	3	32	95	109	65		3.66	0.96
F	38	106	121	30	9		2.56	0.94

Nuchal								
	1	2	3	4	5		mean	SD
M	10	53	86	90	65		3.48	1.11
F	56	112	91	37	8		2.44	1.01

Orbit								
	1	2	3	4	5		mean	SD
M	4	41	88	96	75		3.65	1.04
F	31	81	121	56	15		2.81	1.01

To assess the levels of sexual dimorphism further within the four populations, the groups were assessed separately for differences between their males and females.

Overall, Indian males were more robust than their female counterparts, with the average mean score for males at 3.545 across all four variables, whereas the females had an average mean score of 2.205 across all four variables (Table 5). The males were more variable in their scores, with the standard deviations ranging from 0.93 (mastoid process) to 1.13 (glabella), while the females had standard deviations ranging from 0.55 (glabella) to 0.94 (nuchal crest) (Table 5). Similar to the Indian South Africans, the Coloured South African males were also overall more robust than their female counterparts, with score means from 3.22 (nuchal crest) to 4.03 (supra-orbital margin), while the female score means ranged from 1.38 (glabella) to 3.21 (supra-orbital margin). For three of the four traits, the males were also more variable than the females, with the females only displaying more variation for the supra-orbital margin trait (sd = 1.09; Table 5). Once again, the males were generally more robust among the White South Africans, where score means ranged from 3.82 (mastoid process) to 3.95 (nuchal crest), while the female score means ranged from 1.5 (glabella) to 2.86 (mastoid process). The White South African males were more variable than the females for the glabella and mastoid process traits, while the White South African females were more variable than their male counterparts were for the nuchal crest and supra-orbital margin (Table 5). Black South African males were once again more robust for all four traits when compared to their female counterparts, with score means from 2.83 (nuchal crest) to 3.61 (mastoid process), while the females had score means that ranged from 1.2 (glabella) to 2.8 (supra-orbital) margin. The Black South African males were more variable for the glabella and supra-orbital margin scores, while the females were more variable for the mastoid process and nuchal crest (Table 5).

Of the females, the Black South Africans were the most gracile for the glabella and nuchal crest, while the Indian South Africans were most gracile for the mastoid process and supra-orbital margin. White South Africans were the least gracile for the glabella, while the Coloured South Africans were the least gracile for the nuchal crest (tied with Indian South Africans) and the supra-orbital margin. Of the males, the Black South Africans were the least robust for the glabella and mastoid process, while the Indian South Africans were the least robust for the mastoid process and supra-orbital margin. White South Africans were the most robust for the glabella, mastoid process and nuchal crest, while Coloured South Africans were the most robust for the supra-orbital margin (Table 5).

**Table 5:** Frequencies, means, and standard deviations of scores for each population-sex group for each of the four morphoscopic traits. Abbreviations are defined in table 1

Glabella								
	1	2	3	4	5		mean	SD
BM	8	23	20	18	7		2.91	1.16
BF	65	7	4	0	0		1.20	0.52
CM	6	15	13	28	14		3.38	1.22
CF	55	15	4	2	0		1.38	0.71
IM	2	10	26	15	23		3.62	1.13
IF	41	33	2	0	0		1.49	0.55
WM	2	8	18	20	28		3.84	1.12
WF	45	27	2	1	1		1.50	0.74

Mastoid								
	1	2	3	4	5		mean	SD
BM	0	9	23	33	11		3.61	0.88
BF	14	25	26	9	2		2.47	1.01
CM	1	7	23	24	21		3.75	1.01
CF	8	22	35	9	2		2.67	0.91
IM	1	10	27	28	10		3.47	0.93
IF	12	38	22	4	0		2.24	0.78
WM	1	6	22	24	23		3.82	1.00
WF	4	21	38	8	5		2.86	0.92

Nuchal								
	1	2	3	4	5		mean	SD
BM	4	24	32	13	3		2.83	0.91
BF	31	25	16	3	1		1.92	0.95
CM	5	17	20	24	10		3.22	1.14
CF	6	29	27	11	3		2.68	0.96
IM	0	7	19	22	28		3.93	1.00
IF	5	31	26	11	3		2.68	0.94
WM	1	5	15	31	24		3.95	0.95
WF	14	27	22	12	1		2.46	1.01

Orbit								
	1	2	3	4	5		mean	SD
BM	0	12	26	27	11		3.49	0.93
BF	6	19	37	12	2		2.80	0.89
CM	0	4	21	20	31		4.03	0.95
CF	6	11	29	21	9		3.21	1.09
IM	3	18	27	20	8		3.16	1.03
IF	11	31	26	8	0		2.41	0.87
WM	1	7	14	29	25		3.92	1.00
WF	8	20	29	15	4		2.83	1.04

Pairwise Chi-squared tests showed that the frequencies of the various traits for Black, Indian and White South African males and their female counterparts were significantly different for all four traits, whereas Coloured South African males and females were significantly different from one another for all traits, except for the nuchal crest (*p* = 0.336; Table 6).

**Table 6:** Pairwise chi-squared tests of nominal independence to assess for significant differences in the score frequencies between the males and females of each population group for the four morphoscopic traits. Bold indicates significant differences

Group comparisons		*p*-values^*^			
		glabella	mastoid	nuchal	orbit
Pearson’s Chi-squared		442.64	209.80	257.08	166.79
BM-BF		**< 0.001**	**< 0.001**	**< 0.001**	**< 0.01**
CM-CF		**< 0.001**	**< 0.001**	0.336	**< 0.01**
IM-IF		**< 0.001**	**< 0.001**	**< 0.001**	**< 0.01**
WM-WF		**< 0.001**	**< 0.001**	**< 0.001**	**< 0.001**

* p−value was calculated using a Bonferroni correction factor

When population differences were assessed, the frequencies of the glabella scores demonstrated significant differences (*p* < 0.05) between Black and Indian South Africans, between Black and White South Africans and between Coloured and Indian South Africans. No significant differences were noted between the score frequencies of Black and Coloured South Africans (*p* = 0.642), between the score frequencies of Coloured and White South Africans (*p* = 0.329) and between the score frequencies of Indian and White South Africans (*p* = 1.0; Table 7). For the mastoid process, significant population differences were only noted between Indian and White South Africans (*p* < 0.05), whereas all other groups were not significantly different for the mastoid process score frequencies (Table 7). For the nuchal crest, significant population differences were noted between Black and Coloured South Africans, between Black and Indian South Africans and between Black and White South Africans. No significant differences were noted between Coloured and Indian South Africans (*p* = 0.199), between Coloured and White South Africans (*p* = 0.377) and between Indian and White South Africans (*p* = 0.496; Table 7). For the supra-orbital margin, significant differences were noted between Black and Coloured South Africans, between Coloured and Indian South Africans and between Indian and White South Africans. No significant differences were noted between Black and Indian South Africans (*p* = 0.152), between Black and White South Africans (*p* = 0.157) and between Coloured and White South Africans (*p* = 1.0; Table 7).

**Table 7:** Pairwise chi-squared tests of nominal independence to assess for significant group differences in score frequencies for the four morphoscopic traits. Bold indicates significant differences between population groups

Group comparisons		*p*-value^*^			
		glabella	mastoid	nuchal	orbit
Pearson’s Chi-squared		37.93	28.19	72.97	61.04
B-C		0.642	1.00	**< 0.001**	**< 0.01**
B-I		**< 0.01**	1.00	**< 0.001**	0.152
B-W		**< 0.01**	0.0672	**< 0.001**	0.157
C-I		**< 0.05**	0.14	0.199	**< 0.001**
C-W		0.329	1.00	0.377	1.00
I-W		1.00	**< 0.01**	0.496	**< 0.001**

* p−value was calculated using a Bonferroni correction factor

As significant differences were noted between most populations for at least some of the traits using the Chi-squared tests, binary logistic regression formulae were created for each population separately. Therefore, in order to obtain the best possible outcome in classification, population affinity needs to be estimated prior to estimating sex using the equations below (Tables 8, 9, 10 and 11). However, as population affinity estimation reference data for Indian South Africans is currently not available, a binary logistic regression formula for sex estimation based on the entire sample was also created (Table 12).

To estimate the sex of an unknown individual, the score given for each of the traits is multiplied by the corresponding coefficient (obtained using binary logistic regression) and the resulting values added together. Finally, the constant is added to the previous sum to calculate a discriminant function score (df score). If the resulting calculated discriminant function score is smaller than zero, the individual is most likely female and if the score is greater than zero the individual is most likely male. To calculate the probability of both male and female group membership, the functions.

$$p\left( f \right) = 1/\left( {1 + {e^{ - df\,score}}} \right)\,and\,p\left( m \right) = 1 - p\left( f \right)$$

can be used with p(f) referring to the probability of female group membership and p(m) referring to male group membership [8]. Probability of group membership provides statistical confidence of the estimate and can provide guidance during reporting [26]. Recent research has shown that estimates with probabilities of less than 0.75 may result in a greater chance of erroneous sex estimates, and as a result should be reported as indeterminate [26]. In this study, overall correct classification rates (i.e. with a probability of group membership greater than 0.50), as well as correct classification rates with a probability of group membership greater than 0.75, were reported.

Using all four morphoscopic traits, sex could be estimated for Black South Africans with a 90.8% correct classification rate and no sex bias. The glabella contributed the most to the equation, while the nuchal crest contributed the least (Table 8). However, when the group membership probability was considered, only 77.0% of the Black South African sample was classified correctly with a probability greater than 0.75 (Table 8).

**Table 8:** Binary logistic regression coefficients, constant, cross-validated correct classification rate, and classification matrix for Black South Africans using all four morphoscopic traits

Black South Africans						
	coefficient	z value	Pr(>|z|)	constant	% correct^*^	% correct^**^
glabella	1.8972	5.169	< 0.001	-9.4953	90.8%	77.0%
mastoid	0.7621	2.674	< 0.01			
nuchal	0.6756	2.390	< 0.05			
orbit	0.6778	2.135	< 0.05			

Classification matrix						
	M	F	% correct	M	F	% correct^**^
M	69	7	90.8%	54	22	71.1%
F	7	69	90.8%	13	63	82.9%

*K−fold cross−validated percent correct

**Probability of group membership greater than 0.75

Using all four variables, sex could be estimated for Coloured South Africans with an 88.2% correct classification rate and only a slight female sex bias. Once again, similar to the equation for Black South Africans, the glabella trait contributed the most to the equation; however, the glabella only contributed slightly more than the mastoid process trait. When group membership was considered, correct classifications decreased to 80.3% (Table 9). Only two of the four traits were shown to be significantly different between coloured South African males and females when sex was estimated using binary logistic regression. Therefore, a further equation was created including only the variable scores that showed significant sex differences (glabella and mastoid process scores; Table 10). Correct classification rates increased slightly to 88.8%; however, when only group membership probability of greater than 0.75 was considered, the correct classification rate was slightly lower than for the four-variable model (79.6%).

**Table 9:** Binary logistic regression coefficients, constant, cross-validated correct classification rate, and classification matrix for Coloured South Africans using all four morphoscopic traits

Coloured South Africans						
	coefficient	z value	Pr(>|z|)	constant	% correct^*^	% correct^**^
glabella	1.6042	5.679	< 0.001	-10.0136	88.2%	80.3%
mastoid	1.0815	3.619	< 0.001			
nuchal	0.4555	1.733	0.0832			
orbit	0.4533	1.595	0.1107			

Classification matrix						
	M	F	% correct	M	F	% correct^**^
M	66	10	86.8%	60	16	79.0%
F	8	68	89.5%	14	62	81.6%

*K−fold cross−validated percent correct

**Probability of group membership greater than 0.75

**Table 10:** Binary logistic regression coefficients, constant, cross-validated correct classification rate, and classification matrix for Coloured South Africans using two morphoscopic traits

Coloured South Africans						
	coefficient	z value	Pr(>|z|)	constant	% correct^*^	% correct^**^
glabella	1.674	5.978	< 0.001	-7.305	88.8%	79.6%
mastoid	1.1397	4.003	< 0.001			

Classification matrix						
	M	F	% correct	M	F	% correct^**^
M	67	9	88.2%	57	19	75.0%
F	8	68	89.5%	12	64	84.2%

*K−fold cross−validated percent correc

**Probability of group membership greater than 0.75

The binary logistic equation for the Indian South Africans was able to estimate sex with a correct classification rate of 94.1%, which is the highest of the four South African population groups assessed. Similar to Coloured South Africans, a slight female sex bias was noted for Indian South Africans. Furthermore, the glabella once again contributed the most to the equation, while the supra-orbital margin contributed the least (Table 11). Similarly, when only group membership probabilities of greater than 0.75 were considered, only 88.8% of Indian South Africans could be classified correctly according to sex.

**Table 11:** Binary logistic regression coefficients, constant, cross-validated correct classification rate, and classification matrix for Indian South Africans using all four morphoscopic traits

Indian South Africans						
	coefficient	z value	Pr(>|z|)	constant	% correct^*^	% correct^**^
glabella	2.5998	4.491	< 0.001	-17,3249	94.1%	88.8%
mastoid	1.4532	2.847	< 0.01			
nuchal	1.3475	3.255	< 0.01			
orbit	1.0820	2.092	< 0.05			

Classification matrix						
	M	F	% correct	M	F	% correct^**^
M	70	6	92.1%	67	9	88.2%
F	3	73	96.1%	8	68	89.5%

*K−fold cross−validated percent correct

**Probability of group membership greater than 0.75

The binary logistic equation for the White South Africans was able to estimate sex with a correct classification rate of 91.5% (Table 12). However, similar to the equation for Coloured South Africans, the binary logistic regression equation for White South Africans indicated that only two cranial traits were significantly different between the sexes (glabella and nuchal crest). Therefore, a second logistic regression equation was created for the two variables and resulted in the same correct classification rate of 91.4% (Table 13). Similarly, the correct classification rates when only probabilities of greater than 0.75 were considered were the same for both equations (79.0%; Tables 12 and 13).

**Table 12:** Binary logistic regression coefficients, constant, cross-validated correct classification rate and classification matrix for White South Africans using all four morphoscopic traits

White South Africans						
	coefficient	z value	Pr(>|z|)	constant	% correct^*^	% correct^**^
glabella	1.7433	4.668	< 0.001	-7.2109	91.5%	79.0%
mastoid	0.3857	1.313	0.1891			
nuchal	0.7739	2.633	< 0.01			
orbit	0.4820	1.590	0.1119			

Classification matrix						
	M	F	% correct	M	F	% correct^**^
M	66	10	86.8%	61	15	80.3%
F	3	73	96.1%	17	59	77.6%

*K−fold cross−validated percent correct

**Probability of group membership greater than 0.75

**Table 13:** Binary logistic regression coefficients, constant, cross-validated correct classification rate and classification matrix for White South Africans using two morphoscopic traits

White South Africans						
	coefficient	z value	Pr(>|z|)	constant	% correct^*^	% correct^**^
glabella	1.8784	5.267	< 0.001	-7.2109	91.5%	79.0%
nuchal	0.8104	3.016	< 0.01			

Classification matrix						
	M	F	% correct	M	F	% correct^**^
M	68	8	89.5%	59	17	77.6%
F	5	71	93.4%	15	61	80.3%

*K−fold cross−validated percent correct

**Probability of group membership greater than 0.75

When sex was estimated using random forest modelling, the classification accuracies ranged from 88.0 to 95.7%, with a similar pattern to the binary logistic regression results where Coloured South Africans obtained the lowest and Indian South Africans obtained the highest classification accuracies. Females classified better than males for all groups, except for Indian South Africans, where both males and females classified equally well. The correct classifications rates were similar between the methods for all groups, except for the Indian South Africans for which the accuracy increased slightly with random forest modelling (Table 14). When group membership probabilities of greater than 0.75 are considered for binary logistic regression equations, correct classification rates are substantially lower than those obtained using random forest modelling.

**Table 14:** Classification using random forest modelling. The predictive values and accuracies represent the classification of a separate test samples into male and female groups

	Female predictive value	Male predictive value	*p*-value	κ	Accuracy
Black SA	91.7%	88.9%	< 0.001	0.8056	90.5%
Coloured SA	89.7%	85.7%	< 0.001	0.7537	88.0%
Indian SA	95.7%	95.7%	< 0.001	0.9130	95.7%
White SA	91.7%	90.9%	< 0.001	0.8258	91.3%

*All population groups combined

## Discussion

Sexual dimorphism in the cranium is highly population-specific and making use of standards based on a reference population unrelated to the individual being classified leads to decreased accuracies and probabilities [3]. Therefore, in order to obtain the best possible outcome, population-specific standards need to be created [5–7]. The results of this study indicate that the various South African populations are all highly sexually dimorphic and are able to classify according to sex with high correct classification rates using cranial morphoscopic traits. The highest correct classification rates were obtained for Indian South Africans. Similar, although slightly lower, accuracies were obtained in studies from India, which provided correct classification rates of 85.5–88.7% for sex estimation from the cranium and mandible [27–30]. Enhanced environments and dietary conditions can cause an increase in the degree of sexual dimorphism within a population, whereas unfavourable conditions can cause a decrease [31–35]. Indian South Africans, while segregated during *Apartheid*, were considered semi-privileged or as a part of the trader class, indicating that living conditions were most likely not completely unfavourable [36]. Furthermore, many contract workers that came to South Africa from India in the 1860s, when given the option to stay after their contract expired, chose to remain in South Africa. Their choice was most likely heavily influenced by the possibility of a lower socioeconomic status in India [37]. Therefore, potentially better living conditions in South Africans compared to those in India at the time could explain the difference in sexual dimorphism between the South African Indian and the Indian population in India.

The current accuracies, when a probability of greater than 0.5 indicated a correct classification, were similar or higher than previous research estimating sex from the crania of Black, Coloured and White South Africans [1, 3, 38]. The classification rates for those South African populations, although similar to Indian South Africans, were slightly lower. Coloured South Africans obtained the lowest correct classification rates (88.8%) for morphoscopic sex estimation. Previous research assessing the cranial variation among Black, Coloured and White South Africans also noted lower sexual dimorphism for Coloured South Africans [38]. For the most part males tend to be more robust than their female counterparts [6, 39, 40], which is also true for the current research. However, the degree of sexual dimorphism within a group is affected by genetics among other factors [34, 41]. Due to the extensive heterogeneity of the Coloured South African population [38], overlap between the males and females is present and therefore, most likely resulted in the decreased correct classification rates.

When a probability of greater than 0.75 was considered as the cut-off point for correct classification, the rates for Black, Coloured and White South Africans decreased considerably (by 9.2–13.8%). In contrast, the correct classification rate for Indian South Africans only decreased by 5.3%, most likely due to greater amount of sexual dimorphism between Indian South African males and females when compared to the dimorphism within Black, Coloured and White South Africans.

The newly created binary logistic regression correct classification rates for the morphoscopic traits in Black (90.8%) and White (91.5%) South Africans were higher or similar to those obtained previously [3]. The correct classification for Black South Africans increased by 5.8% from the 2015 study; however, only glabella and mastoid scores were included in the previous equation, whereas glabella, mastoid, nuchal and orbit were included in the current analysis. The additional traits most likely added more information that was able to better separate the males and females during classification.

In contrast to the classification rates for Black South Africans, the correct classification rate for White South Africans only decreased by 1.5% from the previous research, even though both analyses used the glabella and nuchal scores [3]. The White South African females obtained slightly higher trait scores (i.e. were slightly more robust) for the glabella and nuchal traits in the current study, potentially resulting in the marginal increase in misclassifications from the previous equations. Scoring variation between bone and CT scans may have also affected the score frequencies. Previous research testing the repeatability of scoring on bone and on 3D models noted slightly higher repeatability between observers on the 3D models than on the dry bone [42], which was also noted for the current study when compared to the previous research by Krüger and colleagues [3].

Furthermore, the 2015 study made use of samples from the Pretoria Bone Collection that are temporally older than the current sample [2]. While the effect of secular trends was not tested in the current study, secular trends are often the cause of a shift in the degree of sexual dimorphism in a population; however, the shift is typically towards a decrease in sexual dimorphism [43]. Another potential reason for the improved sexual dimorphism noted in the Black South African sample in this study is the origin of the data collected. Previous studies on Black South African cranial variation (sexual dimorphism and population variation) assessed crania from the Pretoria Bone Collection. The collection houses the skeletal remains of individuals from mostly low socio-economic standings. A large proportion of the CT scans used in the current study were collected from the Inkosi Albert Luthuli Central Hospital, which is located in an urban, high- to middle-income area [44]. The potentially higher socio-economic standing of patients scanned at the hospital may have influenced the level of sexual dimorphism present in the Black South African sample.

While the scoring procedure could have influenced the sexual dimorphism present in the White South African sample, another potential cause for the disparity is an actual decrease in the degree of sexual dimorphism between White South African males and females. Again, secular trends may have had an effect on the differences noted between the males and females. Regional variation or changes in socioeconomic status are less likely responsible for the differences, as the samples for both the Krüger et al. [3] study and the current research consisted of individuals from the Gauteng province and individuals with most likely low socio-economic statuses.

## Conclusion

This research is the first to explore cranial sexual dimorphism of modern Indian South Africans when compared to the other South African populations, and provides new information on the current variation present among Black, Coloured and White South Africans in four cranial morphoscopic traits. As a result, new standards were created to estimate sex through morphoscopic analysis with high correct classification rates. The assessment of current Indian South Africans as well as the exploration of the cranial variation present in the other three larger current South African populations, was only possible through the use of 3D cranial models created from head CT scans, and was able to provide novel information for potential future application in both biological and forensic anthropology. However, further research may provide additional information regarding the disparities between the current results and those from previous research. Potential factors that may have influenced the differences between the current and previous research [3] include scoring of 3D models versus scoring of dry bone (for the glabella, mastoid process, nuchal crest and supra-orbital margin), potential secular trends in sexual dimorphism and the potential effect of regional variation in the sample when compared to the previous study. While the cranium is not the most sexually dimorphic skeletal element, many forensic cases in South Africa consist of crania only, and as such, methods that are able to provide additional information on the potential sex of the unknown individual, with probabilities and accuracy rates, are valuable in forensic laboratories in South Africa.
